# Self-stigma and medication adherence among patients with mental illness treated at Jimma University Medical Center, Southwest Ethiopia

**DOI:** 10.1186/s13033-020-00391-6

**Published:** 2020-07-29

**Authors:** Eba Abdisa, Ginenus Fekadu, Shimelis Girma, Tesfaye Shibiru, Temesgen Tilahun, Habib Mohamed, Aaga Wakgari, Amsalu Takele, Milkias Abebe, Reta Tsegaye

**Affiliations:** 1 Department of Psychiatry, School of Nursing and Midwifery, Institute of Health Sciences, Wollega University, Nekemte, Ethiopia; 2 Clinical Pharmacy Department, School of Pharmacy, Institute of Health Sciences, Wollega University, P.O Box 395, Nekemte, Ethiopia; 3 Department of Psychiatry, College of Health Science, Jimma University, Jimma, Ethiopia; 4 Department of Pediatrics and Child Health, Wollega University Referral Hospital, Nekemte, Ethiopia; 5 Department of Obstetrics and Gynecology, School of Medicine, Institute of Health Sciences, Wollega University, Nekemte, Ethiopia; 6 Department of Surgery, School of Medicine, Institute of Health Science, Wollega University, Nekemte, Ethiopia; 7 Medical Microbiology Unit, Department of Medical Laboratory Science, Institute of Health Sciences, Wollega University, Nekemte, Ethiopia; 8 Department of Nursing, Institute of Health Sciences, Wollega University, Nekemte, Ethiopia

**Keywords:** Self-stigma, Mental illness, Medication adherence, Psychiatry, Ethiopia

## Abstract

**Background:**

Self-stigma associated with mental illness has remained a global public health issue affecting social interactions, health care, productivity and acceptance among others. It is one of important factors contributing to non-adherence to medication that leads to increased hospitalization and higher healthcare costs. Hence, the study aimed to assess self-stigma and medication adherence among patients with mental illness treated at the psychiatric clinic of Jimma University Medical Center (JUMC).

**Methods:**

A cross-sectional, community-level study was conducted at Jimma town. The patient’s data was collected from records between April and June 2017 and the collected data was analyzed using SPSS version 21. The Internalized Stigma of Mental Illness (ISMI) tool was utilized to measure internalized stigma. Linear regression analysis was performed to get the final model. Statistical significance association was considered at p-values less than 0.05 and 95% confidence interval was used.

**Results:**

Males comprised more than half (61%) of the total sample of 300 respondents and with a mean age of 34.99 (SD ± 11.51) years. About one-third (32%) of patients had a working diagnosis of schizophrenia followed by major depressive disorder (24.3%). More than half of them, 182 (60.7%) were adherent to their psychotropic medication. The overall mean value of self-stigma was 2.16 (SD = 0.867) and 84 (28%) of the respondents had moderate to high self-stigma. Using ISMI the mean score of alienation was 2.26 (SD = 0.95), stereotype endorsement 2.14 (SD = 0.784), perceived discrimination 2.18 (SD = 0.90), social withdrawal 2.10 (SD = 0.857) and stigma resistance 2.11 (SD = 0.844). Increasing age of the patients (std. β = − 0.091, p = 0.009) and living with kids and spouse (std. β = − 0.099, p = 0.038) were negatively associated with self-stigma whereas increased world health organization disability assessment schedule (WHODAS) score (β = 0.501, p < 0.001), number of relapses (std. β = 0.183, p < 0.01) and medication non-adherence (std. β = 0.084, p = 0.021) were positively associated with self-stigma.

**Conclusion:**

The study revealed that there was high self-stigma among patients with mental illness and a significant association between overall ISMI score and level of medication adherence. These require mental health professionals and policy-makers should give attention to ways to overcome self-stigma and increase medication adherence among patients with mental illness.

## Background

Stigma refers to a social phenomenon whereby the public has a negative view of individuals with attributes perceived by the general population as inferior, threatening, or having other negative connotations [1]. The stigma of psychiatric patients is a term that applies to labeling patients as different and inferior while discrimination is a behavioral manifestation of stigma and omission of patients from certain competitive areas, working as a form of intangible control over groups of people with mental illness [2]. Self-stigma (internalized stigma) refers to the process by which individuals with mental illness apply negative stereotypes to themselves, expect to be rejected by others, and feel alienated from society [3]. It is a gradual process in which a person (example, a psychiatric patient) is uncritically adopting negative societal prejudices about attributes discredited by others [1, 4]. It is related to accepting public stigma in adherence to the concepts of stigma to sustain his/her life [5–7].

Self-stigma associated with mental illness has remained a global public health concern over the past few years [8]. The majority of studies on self-stigma among individuals with mental illness originated from developed nations revealing that the range of self-stigma among patients with mental illness varies from 21.6 to 69% [4, 9–13]. It is associated with psychiatric illness and the main obstacle for preventing early and successful recovery [14]. People with mental illnesses are among the most stigmatized groups in society [15].

Self-stigma affects the quality of life in several ways [5, 16]. As a consequence of self-stigma, patients with mental illness usually have social, psychical, economical and psychological problems. This affects social interactions, health care, productivity and acceptance among others [16–19]. It can manifest in the loss of friends and loved ones—the people most critical to one’s social support network. It can limit employment, housing and educational opportunities [20, 21]. The negative effects of stigmatizing attitudes toward people with mental illness can influence all life domains: living, learning, working and establishing friendships [22, 23]. It worsens mental illness and leads to social exclusion, inability to participate in important life activities, and poor tendency to seek treatment which at last hampers one major dimension of quality of life [5, 16]. It was evident that an individual expecting rejection or condemnation by others tends to be socially withdrawn [1, 23].

Self-stigma is a barrier to the appropriate treatment and rehabilitation for mentally ill patients to avail treatment early that results in prolonged recovery, experience different psychological complications and face serious financial difficulties [13, 22, 24]. Senses of shame, low self-efficacy, and lack of confidence make patients try to avoid stigma by not seeking the required treatment [22, 23]. Outpatients who have adopted prejudices about psychiatric patients have less belief that their mental state will improve, are more depressed and show more negative self-esteem [1, 23, 25]. It is also the leading cause for the reason for more than an average of patients do not seek care, and leaving millions of individuals in the service gap [11, 14, 26–28]. The stigma of severe mental illness also exacerbates the patient burden caused by the illness [14].

Medication non-adherence is a major public health problem that has been called an “invisible epidemic” [29]. Researchers have indicated that self-stigma is one of the potentially important factors contributing to non-adherence to medication. This leads to increased hospitalization, higher healthcare costs and is a predictor of poor outcomes like more relapses, suicide and overall mortality [30, 31]. Self-stigmatization has also a significant influence on medication adherence attitude for patients according to findings from a recent study [32]. Non-adherence to psychiatric treatment regimens has a profound impact on the disease course, relapse, future recovery, cost of health care, and the outcome for the patient [33]. Fear of stigma connected to the diagnosis and fear of rejection due to revealing symptoms have been suggested to further increase non-adherence [34, 35].

Individuals with mental illness have the dual burden of coping with the symptoms of mental illness, like hallucinations, depression, delusions and anxiety, as well as the societal stigmatization of their illness [22, 23]. Stigma results from a process by which certain individuals (groups) unjustifiably are rendered shameful, excluded and discriminated [5, 6]. Self-stigma among people with mental illness can also result from multiple cognitive and environmental factors and processes [36]. Efforts to remove or reduce stigma are still in their infancy [37]. The stigma of mental illness continues to be strong and pervasive in our society and to have detrimental effects on people with mental illness. Overall, stigma impedes recovery from mental illness and, thus, represents a tremendous burden on people with mental illness [24]. Hence, the study was aimed to assess self-stigma and medication adherence among patients with mental illness treated at the psychiatric clinic of Jimma University Medical Center (JUMC).

## Methods

### Study setting, design and eligibility criteria

The study was conducted at the community level in Jimma town patients with mental illness. Jimma town is the capital city of Jimma zone which was founded in the late 1830s and it occurs at south west part of Ethiopia at a distance of 352 km southwest from Addis Ababa. The town has 3 districts (Woreda) and 17 sub-districts (kebele). The town has two hospitals; Jimma general hospital and Jimma University Medical Center (JUMC). Psychiatric outpatient and inpatient services are available only at the JUMC. There were more than 1200 individuals who have ever follow-up treatment at psychiatric clinics of JUMC with different diagnosis and duration of treatment. A cross-sectional study was conducted by reviewing patients’ charts from April 20 to June 20, 2017. Adult patients (≥ 18 years) with mental illness who were listed under the DSM-IV-TR manual and had a history of at least one-time psychiatric treatment at the psychiatric clinic of JUMC were included. Patients who were newly diagnosed with mental illness within 6 months duration before data collection, who were not available during data collection and those who had hearing problems were excluded from the study.

### Study variables

The outcome or dependent variable was self-stigma and the primary independent exposure variable was the level of medication adherence. Other were sociodemographic characteristics (age, sex, place of residence, education, occupation, monthly household expenditure, marital status, living condition, religion), various psychiatric illness’ (bipolar disorder, major depressive disorder, schizophrenia, brief psychotic disorder, schizophreniform disorder, schizoaffective disorder, delusional disorder, panic disorder, generalized anxiety disorder, post-traumatic stress disorder, acute stress disorder, social phobia etc.), duration of treatments, premorbid occupational status, the status of functional outcome, duration of the illness, age at onset of the illness, number of relapses, number of admissions, level of medication adherence and social support.

### Sample size and sampling technique

Single population proportion formula was used to calculate sample size using; the level of significance taken as 95%, (Zα = 1.96), the error of margin, 5% and p = 0.2512%, the previous finding reported that the self-stigma prevalence was 25.12% [36].

$${\text{n}} = \frac{{({\text{Z}}_{{\frac{\upalpha}{2}}} )^{2} {\text{p}}\left( {1 - {\text{p}}} \right)}}{{{\text{d}}^{2} }}$$

where P = extent of self-stigma, Z = critical value at 95% CI of certainty (1.96). d = the margin of error, 5%. n = the required sample size.

Therefore, the sample size was

$${\text{n}} = \frac{{(1.96)^{2} \left( {0.25} \right)\left( {1 - 0.75} \right)}}{{\left( {0.05} \right)^{2} }},{\text{ n}} = 2 8 9$$

Therefore, the sample size by the above calculation was 289. By adding 10% non-response rate, the final sample size was 318.

For obtaining study population, a systematic sampling method was used because the registration of the patients with mental illness who were only from Jimma town was used. Patients’ data registration revealed that the total adult patients with mental illness who had treatment at the psychiatry clinic of JUMC were 1200 and from these 698 adult patients were from Jimma town. From these, 318 study participants were selected systematically by using a computer-generated method (k = 2). By reviewing chart study participants were identified. Finally, the information of adult psychiatric patients was distributed to health extension workers of the town and psychiatry department staff according to the patients’ address. Information of the study sample was provided to the health extension workers who contacted the patients using tracing methods and with the help of the contact number provided on the registration chart. But 14 of them died during data collection. Therefore, they were excluded from the study and data collection and analysis was carried out among 304 participants.

### Data collection tools and process

The data was collected from patients’ charts and using interview-based structured questionnaires among the participants who were addressed by tracing methods in each kebele of the town. Two Bachelor of Science (B.Sc) psychiatric nurses and four mental health master’s students were recruited for data collection.

The data collection tool had five components: socio-demographic variables, clinical details, self-stigma of mental illness (ISMI), WHODAS version 2 (12-items), Oslo scale for social support and Morisky Medication Adherence Scales (MMAS-4). Clinical inventory part includes patients’ working diagnosis, age at onset, duration of illness, number of episodes, number of hospitalizations, duration of treatment, current clinical status, medication side effects, and previous suicide attempt.

The Internalized Stigma of Mental Illness (ISMI-29 items) tool [13, 38–40] was utilized to measure internalized stigma (internal consistency reliability coefficient of alpha = 0.90) [41]. The tool was validated and has been used in several studies in Ethiopia [5, 36, 42]. Each ISMI item contains a declarative statement about a potential stigma issue and participants respond to each statement by indicating their level of agreement: 1 = strongly disagree; 2 = disagree; 3 = agree and 4 = strongly agree. It contains five subscales; alienation (6 items), stereotype endorsement (7 items), discrimination experience (5 items), social withdrawal (6 items), and stigma resistance (5 items). Alienation is “the subjective experience of being less than a full member of society”. The stereotype endorsement is “the degree to which patients agreed with common stereotypes about people with a mental illness”. The discrimination experience measures “respondents’ perceptions of the way they tend to be treated by others”. The social withdrawal measures the self-exclusion from social events (situations) due to mental illness. The stigma resistance subscale is “a person’s ability to resist stigma” [16, 36]. Except for the stigma resistance domain, a higher score of the remaining four subscales indicates higher internalized stigma. For that matter, stigma resistance items were reverse coded. The overall score was obtained by summing all the answered scores and divided by a total number of items.

The other tool which was developed by the World Health Organization, The 12-item Self-Report World Health Organization Disability Assessment Schedule 2.0 (WHODAS-II) [43, 44] was used to establish the level of impairment associated with mental illness and it assesses the level of disability and the number of days lost from work in the previous 30 days. It has 12 items and Likert scale (1–5). Oslo scale for social support has three items and is used to assess the level of social support of the participants [45, 46]. Oslo-3 Social Support Scale (OSS-3); the interpretation was made as, a person scored 12–14 (good), 9–11 (moderate), and 3–8 (poor social support) [47].

The Morisky Medication-taking Adherence Scale (MMAS-4), a four-item self-report measure of medication-taking behavior, which has high reliability and validity, was used for assessing patient adherence [48]. MMAS-4 was reliable in the study (Cronbach’s α = 0.73) done in Jimma university specialized [41]. It measures both intentional and unintentional adherence based on forgetfulness, carelessness, stopping medication when feeling better, and stopping medication when feeling worse. Each of the items has dichotomous types of responses (yes, no). In all, 0 point was allocated to a yes response and 1 point to a no response. The total score ranged from 0 (non-adherent) to 4 (adherent). The results were scored for all questions of medication adherence with a score of ≤ 2 classified as ‘non-adherent’ and > 2 as adherent [39, 41].

### Data quality management and analysis

To ensure the quality of data, the project staff were trained for 2 days on the purpose of the study, details of the questionnaires, the technique of interview, the importance of privacy and confidentiality of the respondents. The data collection instrument tools were developed in English version, then translated to local language (Amharic and Afan Oromo) and later translated back to English version by experts for consistency. Twenty patients were pretested at the psychiatric clinic, JUMC, those who came for appointments from different parts of Jimma Zone except Jimma Town. During data collection process, all the collected data was reviewed daily and checked for completeness.

The collected data were entered into Epi-data version 3.1, and then exported to SPSS window version 21 for summarization and further analysis. Before analysis linear regression assumptions were checked. Simple linear regression analysis was carried out to assess the association between different variables and to identify candidates for multivariate linear regression analysis. Variable having p-value of less than 0.25 was subjected to multivariate linear regression analysis. Then, multivariate linear regression analysis was performed to get the final model. Statistical significance association was considered at p-values less than 0.05 and 95% confidence interval was used. Self-stigma was identified against medication adherence status. The mean score for each of the subscale and overall self-stigma was done but the final subscale of ISMI was reversely recoded before the identified mean score. Finally, the results were presented in tables, graphs and statements.

### Operational definition

*Level of stigma* was based on the mean score of ISMI, and using similar score categories to the European and Ethiopian studies [39, 42]:

- < 2 of total score indicated that minimal internalized stigma.
- 2–2.5 of total score indicated that low internalized stigma.
- 2.5–3 of total score indicated that moderate internalized stigma.
- 3+ of total score indicated that high internalized stigma.

*Adherence to medication* The level of drug adherence was measured based on MMAS-4 score. Accordingly,

- Non-adherent: MMAS-4 score of ≤ 2.
- Non-adherent: MMAS-4 scores of > 2.

## Results

### Socio-demographic characteristics

Of the total 318 participants contacted by tracers, 14 people died during the study period. Only 304 participants were included in the study with response rate of 300 (98.67%). Of the total respondents, males comprised 185 (61.7%) and the mean age of the respondents was 34.99 (SD = 11.51) years. One hundred and eighty-five (61.0%) were single and the majority of them were Muslims (43.0%). One hundred forty-five (48.3%) of them were Oromo, and 192 (64.2%) of them were living with their family. One hundred forty (46.7%) of the respondents were jobless and about one-third (34.3%) had primary educational level (Table 1).

**Table 1:** Sociodemographic variable of the sample (n = 300)

Socio-demographic characteristics	Frequency (n)	Percent (%)
Sex		
Male	185	61.7
Female	115	38.3
Marital status		
Single	180	60.7
Married	80	26.7
Divorced	22	7.3
Widowed	11	3.7
Separated	5	1.7
Religion		
Muslim	129	43.0
Orthodox	121	40.3
Protestant	44	14.7
Catholic	4	1.3
Others^a^	2	0.7
Ethnicity		
Oromo	145	48.3
Amhara	75	25.0
Dawuro	28	9.3
Gurage	21	7.0
Kafa	19	6.3
Others^b^	31	4.0
Living condition		
With family (father or mother)	194	64.7
With wife/husband and kids	62	20.7
Living alone	17	5.7
With relatives/friends	12	4.0
Living on street	5	1.7
Others^c^	8	2.7
Occupation		
Jobless	139	46.3
Employed	54	18.0
Student	29	9.7
Merchant	26	8.7
House wife	23	7.7
Daily laborer	16	5.3
Retired	7	2.3
Farmer	6	2.0
Educational status		
Illiterates	19	6.3
Able to read/write, but no formal education	6	2.0
Primary	103	34.3
Secondary	89	29.7
College and above	83	27.7

^a^ Catholic, Wakeffata

^b^ Yem, Kefa, Sidamo, Wolayita

^c^ In prison, dormitory

### Clinical characteristics

About one-third (32%) of patients had a working diagnosis of schizophrenia followed by major depressive disorder (24.3%). The average duration of mental illness among the respondents was 98.25 (SD = 67.608) months ranged from 3 to 12 months. The mean value of duration before seeking treatment was 14.28 (SD = 24.31) months. The average duration that respondents had been getting treatment was 69.11 (SD = 64.98) months, ranged zero to 348 months. After the first episode of their illness, respondents experienced an average value of 2.17 (SD = 2.511) times relapse of their illness with a maximum of 17 times. The average number of admissions to the hospital was 0.87 (SD = 1.289) with the range of no admission to seven times. The mean age of the participants at the onset of mental illness was 26.90 (SD = 11.484) years.

Around 74.7% of the respondents were on the work (had jobs) and around two-thirds (65.7%) of them were single when they had the onset of mental illness. Less than two-thirds of the respondents (63.3%) had a history of discontinuation of their medication without medical advice. Fifty-five (18.3%) respondents had poor social supports whereas 39 (13.0%) of them had high social supports. About 30.7% of respondents had a history of attempting suicide to kill themselves with and without strong intentions. About 13% of the respondents attempted suicide due to their illness alone whereas 6.8% of them attempted due to their illness comorbid with social problems like lack of support. More than two-thirds (69.3%) of the respondents had current follow up from psychiatric clinics, but only 63.3% of the respondents were on treatment during the data collection period (Table 2).

**Table 2:** Clinical diagnosis of the sample (n = 300)

Diagnosis	Frequency (n)	Percent (%)
Schizophrenia	96	32.0
Major depressive disorder	73	24.3
Bipolar disorder	58	19.3
Brief psychotic disorder	20	6.7
Schizophreniform disorder	14	4.7
Schizoaffective disorder	12	4.0
Post-traumatic disorder	7	2.3
Generalized anxiety disorder	6	2.0
Delusional disorder	5	1.7
Others^a^	9	3.0

^a^ Dementia, social phobia, somatoform disorder, sleep disorder, substance disorder

### Level of medication adherence and self-stigma

Medication adherence was assessed using the Morisky medication adherence scale (MMAS) which contains four components. With this scale, 182 (60.7%) were adherent to their medication and 39.3% were not-adherent. From 39.3% of the respondents who were non-adherents, 26% of them were males. Those individuals forgetting taking of their medication at right time and right dose were 127 (42.3%), careless in taking their medication 106 (35.3%), discontinued their medication when felt better 88 (29.3%) and those who stopped their medication when their illness becomes worse were only 52 (17.3%).

The self-stigma was evaluated using the ISMI scale that contains 29-items with four-point Likert scale and has five subscales. The mean score of each sub-scale was done with different tools. Accordingly, mean alienation score 2.26 (SD = 0.95), mean stereotype endorsement score 2.14 (SD = 0.784), mean perceived discrimination score 2.18 (SD = 0.90), mean social withdrawal score 2.10 (SD = 0.857) and mean stigma resistance score 2.11 (SD = 0.844). The stigma resistance component was reversely recoded because the maximum value indicates that resistance is low in opposite of other components of IMSI. The overall mean value of self-stigma was 2.16 (SD = 0.867).

About 157 (52.3%), 59 (19.7%), 42 (14%), and 42 (14%), of the respondents had a minimal, low, moderate and high level of self-stigma based on total stigma score., respectively. Overall, around one-third (28%) of the respondents had moderate to high self-stigma (Table 3).

**Table 3:** The prevalence and overall ISMI score of the sample (n = 300)

ISMI sub-scales and items	Frequency (n)	Percent (%)
Alienation (yes)		
Feeling of worthless	100	33.3
Miserable life due to having mental illness (MI)	127	42.3
Others people don’t understand me	101	33.7
Feeling of shame due to having MI	110	36.7
Feeling bad or annoyed due to having MI	133	44.3
Stereotype (yes)		
Low self esteem	98	32.7
Public misconceptions work on me	102	34.0
People knows that I am mentally ill by just looking at me	90	30.0
PWMI are danger to other	45	15.9
Decisions about you shall be made by others	47	15.7
PWMI will not live better life	59	19.7
PWMI should not have marital life	49	16.3
Perceived discrimination (yes)		
Because of my MI, I can’t do good to the public	49	16.3
Because of my MI other people discriminates me	92	30.7
Others believe that I will not be successful because of my MI	108	36.0
Other people will not give me value/concern because of my MI	95	31.6
Other people treat me as a child or inferior because of my MI	89	29.6
Other people prefer not to approach me or don’t want close relation with me	80	26.7
Social withdrawal (yes)		
Not to bother others about me, am not talk to them about my self	130	43.4
I have no good social relationship due to the fear of behaving bad because of my MI	100	33.4
Misconception from the public made me isolate my self	88	29.4
I don’t feel good in a public place	89	29.7
Avoid healthy social contact with people to protect discrimination	80	16.7
For the sake of my family not to feel ashamed because of me, I isolate myself from social relation	80	26.6
Stigma resistance (no)		
Feel comfortable when other see my symptom of MI	55	18.3
I am living a kind of life I want to live	207	46.6
I can live a better life despite my MI	174	58.0
PWMI can contribute well to the public	216	72.0
My MI makes me be stronger in my life	101	53.7

*MI* mental illness, *PWMI* patient with mental illness

### Factors associated with self-stigma

For one by one regression association of each dependent factor with self-stigma around 17 variables were associated and candidate for multivariate linear regression. Medication non-adherence, having diagnosed with schizophrenia, living with others other than parents, increased WHODAS score (decreased functional outcome), stopped their treatment during data collection, having poor social support, increased number of admission, having frequent relapse of the illness and longer duration of the illness before starting treatment were associated with significantly higher self-stigma whereas the remaining variables listed in Table 4 were associated with lower level of self-stigma upon binary linear regression (Table 4).

**Table 4:** Variables associated with self-stigma in the simple regression analysis model of the sample

Model	Coefficients^a^					
	Unstandardized coefficients	Standardized coefficients	T	Sig.	95% confidence interval for β	
	β	β			Lower bound	Upper bound
Medication non-adherence	0.034	0.352	6.486	.000	.057	.117
Bipolar disorder	− 0.032	− 0.092	− 1.600	.111	− .071	.007
Others psychotic disorders	− 0.041	− 0.114	− 1.974	0.049	− .082	.000
Schizophrenia	0.032	0.108	1.877	0.061	− .002	.065
Living with others other than parents	0.032	0.080	1.379	0.169	− .013	.072
Living with their wife and kids	− 0.066	− 0.190	− 3.334	0.001	− .104	− .027
Currently living with spouse	− 0.078	− 0.254	− 4.532	0.000	− .112	− .044
Functional outcome (WHODAS score)	0.003	0.594	12.761	0.000	.002	.003
Having job	− 0.077	− 0.282	− 5.066	0.000	− .107	− .047
Having premorbid marriage	− 0.057	− 0.191	− 3.356	0.001	− .091	− .024
Having premorbid job	− 0.055	− 0.175	− 3.076	0.002	− .090	− .020
Stopping their treatment	0.050	0.177	3.113	0.002	.018	.082
Having strong social support	− 0.098	− 0.241	− 4.289	0.000	− .143	− .053
Having poor social support	0.073	0.208	3.676	0.000	.034	.113
Age at onset of the illness	− 0.002	− 0.153	− 2.666	0.008	− .003	.000
Number of admissions	0.024	0.229	4.060	0.000	.013	.036
Number of relapses	0.019	0.349	6.431	0.000	.013	.025
Duration of the illness before starting treatment	0.001	0.090	1.552	0.122	.000	.001
Number of family members	− 0.006	− 0.104	− 1.799	0.073	− .013	.001
Monthly income	− 2.577E−5	− 0.186	− 3.266	0.001	.000	.000
Age of the patients	− 0.001	− 0.107	− 1.862	0.064	− .003	.000

^a^ Dependent variable: self-stigma

Multivariable regression revealed the age of the patient (std. β = − 0.091, p = 0.009) and living with kids and spouse (std. β = − 0.099, p = 0.038) were negatively associated with self-stigma whereas increased WHODAS score (β = 0.501, p < 0.01), number of relapses (std. β = 0.183, p < 0.01) and medication non-adherence (std. β = 0.084, p = 0.021) were positively associated with self-stigma. Increasing the age of the patients and living with kids and spouses was significantly associated with lower level of internalized stigma whereas the rest variables were associated with higher level of internalized stigma (Table 5).

**Table 5:** Variables associated with self-stigma in the final model of adjusted multivariate linear regression analysis of the sample

	Coefficients^a^					
	Unstandardized coefficients	Standardized coefficients	t	Sig.	95% confidence interval for β	
	β	β			Lower bound	Upper bound
Age of the patient	− .001	− .091	− 1.899	.009	− .002	.000
Number of relapses	.010	.183	3.868	.000	.005	.015
Increased WHODAS score	.002	.501	10.195	.000	.002	.003
Medication non adherence	.023	.084	1.750	.021	.003	.050
Living with kids and spouse	− .034	− .099	− 2.081	.038	− .067	− .002

^a^ Dependent variable: self-stigma and discrimination

### Association of self-stigma and level of medication adherence

By dichotomizing the level of medication adherence into adherent and non-adherent, it was associated with self-stigma. In the bivariate (β = 0.034, p < 0.001) and multivariate linear regression model (std. β = 0.084, p < 0.021), being non-adherent to the medication was associated with higher self-stigma.

Among respondents who were adherent to medications, minimal stigma was detected among 63.2% whereas moderate to high stigma was detected in 17.5%. Of the respondents who were non-adherent to their medication, 44.1% had moderate to high internalized stigma. Among the respondents who were forgetting to take their medication, 26.0% of them had high levels of stigma and nearly half (47.2%) of respondents who were careless in taking of their medication had moderate to high self-stigma. About 37.5% of respondents with a history of medication discontinuation when they felt well from their illness, had moderate to high self-stigma. About 51.9% of the respondents who had a history of medication discontinuation when felt their illness worsen without medical advice had moderate to high self-stigma score (Table 6).

**Table 6:** Adherence status against the self-stigma of the sample

Variables	Stigma level			
	Minimal stigma	Low stigma	Moderate stigma	High stigma
Status of adherence				
Adherent	115 (63.2%)	35 (19.2%)	23 (12.6%)	9 (4.9%)
Non adherent	42 (35.6%)	24 (20.3%)	19 (16.1%)	33 (28.0%)
Total	157 (52.3%)	59 (19.7%)	42 (14.0%)	42 (14.0%)
MMAS-4				
Forgetting their medication	43 (33.9%)	29 (22.8%)	22 (17.3%)	33 (26.0%)
Careless in taking their medication	34 (32.1%)	22 (20.8%)	18 (17.0%)	32 (30.2%)
Discontinue their medication when felt better	38 (43.2%)	17 (19.3%)	14 (15.9%)	19 (21.6%)
Discontinue their medication when felt their illness becomes worsened	16 (30.8%)	9 (17.3%)	9 (17.3%)	18 (34.6%)

*MMAS* Morisky medication adherence scale

**Fig. 1:**
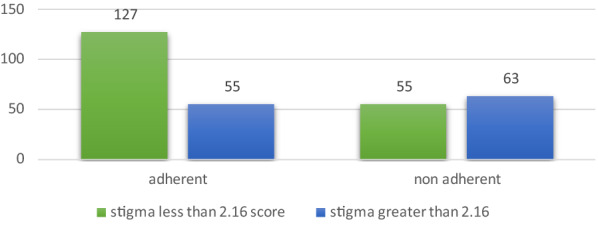
Stigma score around the overall mean score with the level of medication adherence among the sample

Overall, more than half (53.4%) of the respondents who were non-adherent scored above the total mean score of self-stigma and discrimination (Fig. 1).

When comparing the respondents with a history of never forgetting to take their medication, respondents who had a history of forgetting to take their medication, had a higher level of self-stigma (std. β = 0.314, p = 0.002). Additionally, respondents who were careless in taking of their medication had higher self-stigma (std. β = 0.364, p < 0.001) compared to those who were not careless. Stopping to take their medication when they felt well from their illness (std. β = 0.134, p = 0.004) and when felt worse from their illness (std. β = 0.247, p < 0.001) had higher self-stigma compared to the counterparts.

## Discussion

This study tried to address the association between self-stigma and medication adherence among psychiatric patients. Self-stigma is an important factor negatively influencing adherence to treatment and significantly contributing to the voluntary discontinuation of drugs. Cross-cultural studies indicate that every culture has its own particular explanations for health and illness and its healing strategies [49]. Ethiopians commonly believe that mental illness is caused by evil spirits and should be treated with holy water and exorcism. Most Ethiopians have faith in traditional healers and procedures [50]. The possible factors for the high risk of stigma are ignorance, perceived fear of injury, actual (perceived) absence of treatment, and cultural misconceptions about the nature of the mental illness, associating the illness with the supernatural explanation [5–7, 51]. The rural population’s adherence to spiritual explanations, particularly for serious psychotic symptoms, and their openness to both modern and traditional treatments speak to the importance of developing a holistic and inclusive approach to psycho-education and treatment for this population [52]. A clearer understanding of the cross-cultural nature of stigma and discrimination experienced by people living with mental disorders will also be an important avenue for future investigation.

The majority of respondents had working a diagnosis of schizophrenia followed by major depressive disorder. The mean value of time duration for which the respondents had followed their treatment was 69.11 months and more than half of the respondents had history of discontinuation of their medication without medical advice. About 30.7% of the respondents had feeling of socially discriminated against due to their illness.

In this finding, the overall self-stigma score was 2.16 and 28% of the respondents had moderate to high self-stigma. The prevalence of the self-stigma was lower compared with the study done in Jimma [36] and Dilla [5], whereas it was comparable with the study done in Addis Ababa [42]. The first study in Jimma and Dilla were done among patients with mental illness only those on follow up and facility-based, while the current study was done both at community-based and tracing methods. Additionally, the current study also took place among all patients who had a history of treatment at JUMC without limiting the duration and clinical diagnosis.

The prevalence of self-stigma was higher in this study compared with the studies done in Iran, Europe and Nigeria [13, 53, 54]. The study done in Iran took place among patients with bipolar disorders while the study in Nigeria was only among patients with severe mental disorders. Additionally, cross-sectional facility-based study conducted in Addis Ababa, Ethiopia reported that nearly three-quarters of respondents (71%) expressed strong agreement to at least one internalized stigma item [42]. A study by Gebeyehu et al. reported that (25.7%) of patients with severe mental disorders attending Bahir Dar Felege Hiwote Referral hospital had perceived stigma [29]. Additionally, study by Adewuya et al. in Lagos, Nigeria reported that high self-stigma was found in 21.6% of the outpatients in the study [13]. The correlates of high self-stigma study in Nigeria included level of social support, duration of illness, level of insight and working status.

This study found that social withdrawal (exclusion from social events) was less experienced than other components of ISMI whereas alienation (being inferior) was the highest score in agreement with the study done in Jimma, Dilla and Europe that identified high alienation score [5, 36, 54]. But a study in Nigeria revealed that ISMI scoring was highest on ‘dis-crimination experiences’ (mean = 2.69, SD = 1.88), followed by ‘social withdrawal’ (mean 2.40, SD = 1.51) [13].

The increasing age of the patients was significantly associated with a lower level of internalized stigma. But a study by Kamaradova et al. reported the levels of self-stigma expressed by the total ISMI scores did not significantly correlate with either age or age of disorder onset [1]. This leads to more adaptation and creates some relation with the community which may decrease the magnitude of self-stigma.

In our study finding, 55 (18.3%) had poor social supports whereas 39 (13.0%) of them had high social supports. A closely related finding was reported by Gebeyehu et al. where 45.9% had good social support [29]. Living with kids and spouses was negatively associated with self-stigma up on multivariable linear regression. This finding correlates with the previous study by Asrat et al. that showed a lack of family support was independently associated with internalized stigma. Accordingly, patients getting good family support had less risk for internalized stigma than their counterparts [5]. These studies strongly supported that patients getting better social and family support had a better prognosis and favorable outcome against internalized stigma. Furthermore, they are actively involved in anti-stigma campaigns and other activities.

The current study found that 39.7% of respondents were non-adherent with their medication. This finding was in line with global non-adherence rates among patients with mental illness between 30 and 65% [55, 56]. The finding was also comparable with those from Jimma, Ethiopia 41.2% [41], and India 42% [57]. Contrary to this, the prevalence was higher than in India 26% [58] and Addis Ababa, Ethiopia (26.5%) [42]. The finding was also lower than a study conducted in Nigeria (48.0%) [59], Curitiba, Parana (49%) [60], Nigeria 54.2% [61], Bahir Dar, Ethiopia (55.2%) [29], South Africa 63.2% [62] and Pakistan’s study (64.75%) [63]. This variation could be due to the differences in socio-demography of the study population, selection criteria for the study participants, study design, sample size and tools used for the assessment of adherence. It might also relate to the differences in the health care setting, community culture or level of knowledge on drug adherence of health providers.

For example, the lower sample size was taken in Pakistan, using a convenient, non-probability sample of 135 follow-up patients were assessed. The study done in South Africa took only among schizophrenic patients and the study done in Nigeria was carried out at facility-based and among patients struggling with illness a sign and symptoms by using self-administered questionnaires. Adherence at any given moment does not guarantee subsequent adherence since the definition attributed by the people to the use of the drugs and the motivation to do so are flexible, they are influenced by their perception, attitude, external factors, and past experiences. Thus, even adherent patients need great focus and monitoring regarding the use of medication. According to the available evidence, non-adherence to psychiatric medications is high and is one of the top public health problems requiring due attention and intervention [41].

In this study, significant association was identified between self-stigma and levels of medication adherence. Study participants who didn’t adhere to their medication as ordered by health care providers were experiencing more self-stigma than those patients who were adherent. It was in agreement with other research outcomes [5, 64]. One study done in Czech Republic, by placing self-stigma as independent (predictor factors) for medication adherence, identified that there was negative association between self-stigma and medication adherence among schizophrenic patients [1]. Contrary to this, one study done in Sweden identified that there was no significant association between the two factors in the adjusted model [38].

Even though self-stigma and discrimination could occur among all patients, a high mean score of self-stigma was common among non-adherent whereas low mean score was common among adherents than non-adherents. About 39.3% scored greater than the overall mean value of ISMI score. Visiting psychiatric clinics and taking medications are directly associated with the disclosure of their health status. In general, the present study showed that higher self-stigma level was linked to non-adherence. This underlines the importance of interventions aimed at lowering the levels of self-stigma in patients.

Although the treatment gap is a useful construct to measure access and equitability of care, it fails to communicate the real-life consequences of the treatment gap and the urgent need to address care disparities in Ethiopia [65]. Lack of capacity is deeply rooted in the lack of psychiatric training available in Ethiopia. Resources for learning about mental illness fuel the fire of stigma and ignorance as well. Medical journals exist in Ethiopia, but rarely ever broach the topic of mental illness [66]. Stigma has long been viewed as a major barrier to mental health reform and community integration for people with mental disorders. Whereas, knowledge of the social dimensions of stigma and discrimination on mental patients has begun to accumulate, much remains to be known about the extent and models of self-stigma and how to measure it.

## Limitations of the study

One of the study’s limitations is the fact that most data were obtained using self-reported questionnaires. The self-report method used to measure self-stigma and medication adherence might substantially overestimate adherence, as it relies on patient response. To get the study participants, we used a general survey method on patients with mental illness who had a history of treatment at the psychiatric clinic of JUMC which might lead to recall bias and great variability in the information of the current data (can significantly affect the reliability and validity of the result). The cross-sectional design of the study fails to assess patients’ adherence behaviors over time, and although this approach is helpful to investigate associations between variables, it cannot attribute cause and effect. Another limitation of this study pertains to the lack of longitudinal analysis concerning factors associated with self-stigma has been highlighted previously. The study setting and sample were not completely representative of the patient population with mental illness patients. In this study, we assessed only internalized stigma and perceived discrimination but also it would be better if others stigmas and discriminations like social, institutional and applied stigma to identify the magnitude and association with the level of medication adherence among patients with mental illness. Further, this research might not identify all factors (like severity and disease symptomatology) contributing to non-adherence, nor accurately measure adherence, which can be addressed by future studies. Finally, we were unable to address those patients who left the town for different reasons.

## Conclusion

About one-third of the patients had a working diagnosis of schizophrenia. Age, living with kids and spouse, increased WHODAS score, the number of relapses and medication non-adherence were significantly associated with self-stigma. There was a significant association between overall ISMI score and level of medication adherence that indicated becoming non-adherent was associated with a higher level of self-stigma and discrimination. It was identified that there was high self-stigma related to medication non-adherence that leads mental health professionals and policy-makers to give future direction to self-stigma in perspectives of medication adherence. Health professionals in the psychiatric clinic and pharmacists need to focus on and counsel patients about adherence and its implications for their clinical outcome.

Religious leaders could be engaged by marshaling religious teachings that admonish individuals from discriminating against mentally ill persons. The current report, along with other similar data from Ethiopia and other low-income countries, supports the need to incorporate culturally appropriate methods of addressing internalized stigma into rehabilitation packages for this group.

Strategies focusing on early detection and treatment of mental illness will play an important role in stigma prevention. Awareness creation, community mobilization, and service expansion strategies are cost-effective and efficient ways to act against stigma. Further, strengthening the social network (support) of mentally ill people with themselves and with the community is found to be necessary. Working with the media to raise awareness of mental health issues and establish best practices for reporting and for depicting mental illness is an effective approach with the potential to have a positive impact on public perceptions.

## Data Availability

The dataset used and/or analyzed during the current study are available from the corresponding author on reasonable request.

## Abbreviations

ISMI: Internalized stigma of mental illness
JUMC: Jimma University Medical Center
MI: Mental illness
MMAS: Morisky Medication Adherence Scale
OSS-3: Oslo-3 Social Support Scale
PWMI: Patients with mental illness
SD: Standard deviation
WHODAS: World Health Organization Disability Assessment Schedule
